# The T1799A point mutation is present in posterior uveal melanoma

**DOI:** 10.1038/sj.bjc.6604731

**Published:** 2008-11-04

**Authors:** C S Janssen, R Sibbett, F L Henriquez, I C McKay, E G Kemp, F Roberts

**Affiliations:** 1 Division of Infection & Immunity, FBLS, University of Glasgow, Glasgow, UK; 2 Department of Pathology, Western Infirmary, Glasgow, UK; 3 School of Engineering and Science, University of the West of Scotland, Paisley, UK; 4 Sherbrooke Consultants, 22 Sherbrooke Drive, Glasgow, UK; 5 Tennent Institute of Ophthalmology, Gartnavel General Hospital, Glasgow, UK

**Keywords:** melanoma, choroid, ciliary body, cytogenetic, heterogeneity, *BRAF* gene

## Abstract

An activating mutation in exon 15 of the *BRAF* gene is present in a high proportion of cutaneous pigmented lesions. Until recently this mutation had however only been identified in one case of posterior uveal melanoma. Despite this apparent lack of the *BRAF* mutation, inappropriate downstream activation of the Ras/Raf/MAPK pathway has been described in posterior uveal melanoma. Based on the already recognised morphological and cytogenetic heterogeneity in uveal melanoma, we hypothesised that the *BRAF* mutation may be present in uveal melanoma but only in some of the tumour cells. In this study, we analysed 20 ciliary body and 30 choroidal melanomas using a nested PCR-based technique resulting in the amplification of a nested product only if the mutation was present. This sensitive technique can identify mutated DNA in the presence of wild-type DNA. The mutation was identified in 4 of 20 (20%) ciliary body and 11 of 30 (40%) choroidal melanomas. Further analysis of separate areas within the same choroidal melanoma demonstrated that the mutation was not present in the entire tumour. In conclusion, the T1799A *BRAF* mutation is present in a proportion of posterior uveal melanomas but within these tumours the distribution of the mutation is heterogeneous.

Mutations in the *BRAF* gene (a member of the Raf family that encodes a serine/threonine protein kinase) have been shown to occur in the majority of cutaneous melanomas (Brose *et al*, 2002; Davies *et al*, 2002). In particular a single point mutation in exon 15 (T1799A), which results in constitutive kinase activity and unregulated signal transduction, is involved in up to 80% of cases (Brose *et al*, 2002; Davies *et al*, 2002). Despite the high incidence of this mutation in cutaneous melanoma, several studies have shown that it is not present in posterior uveal melanoma (Cohen *et al*, 2003; Weber *et al*, 2003). The *BRAF* mutation has however been identified in several primary uveal melanoma cell lines and recently Malaponte *et al* (2006) identified the mutation in one primary uveal melanoma. Recently, we have identified that the mutation also occurs in iris melanoma (Henriquez *et al*, 2007). The reason for this discrepancy in reporting of the *BRAF* mutation is not clear. Cutaneous and uveal melanoma share a common embryological origin from cells of the neural crest and often exhibit similar histological characteristics (Hogan *et al*, 1971). However, it is well-recognised that individual uveal melanomas display considerable morphological and cytogenetic heterogeneity (COMS, 1998; Sandinha *et al*, 2006; Maat *et al*, 2007). To date the methods used to identify the *BRAF* mutation have not taken this potential genetic heterogeneity into account. The negative results from previous studies may reflect the inability of the technique used to identify the mutation in a minority of cells in a background of cells containing the wild-type *BRAF* gene.

To address this problem we used a nested PCR-based technique, which would result in the amplification of a nested product only if the mutation was present. This technique was used to screen posterior uveal melanoma samples for the presence of the *BRAF* mutation and secondly to examine separate areas within individual tumours to confirm genetic heterogeneity.

## Materials and methods

### Case selection

Archival formalin- or gluteraldehyde-fixed, paraffin-embedded tissue sections from choroidal and ciliary body melanomas were retrieved from the Eye Pathology Files, Western Infirmary Glasgow. Clinical and histological details (such as age, sex, metastasis, largest basal dimension of tumour, cell types, presence of closed vascular loops) were obtained from the pathology report. A total of 30 choroidal melanomas and 20 ciliary body melanomas were selected for this study. This project received the full approval of the West Ethics Committee, North Glasgow Hospitals, and adhered to the tenets of The Declaration of Helsinki. Normal tissue adjacent to the tumour was used as a negative control. The SKmel-28 cell line was used as a positive control. Human foreskin fibroblasts (HFF) were used as a negative control for developing the technique.

### Genomic DNA isolation

One unmounted haematoxylin and eosin- (H&E) stained 4 *μ*m section was examined from each case and appropriate areas of tumour and normal tissue identified. A 15 *μ*l droplet of proteinase K (1% solution in 10 mM Tris (pH 8.5) and 1 mM EDTA) was added to the relevant areas on the section and the cells scraped with a fresh scalpel blade until suspended in the droplet. The solution was then pipetted into a 0.2 ml PCR tube. The samples were incubated at 55°C for 20 h. Following the addition of 15 ml of water, proteinase K was inactivated by incubation at 95°C for 8 min. The isolated DNA was stored at 4°C until further use in PCR. For all choroidal and ciliary body melanomas one area of tumour was sampled for investigation. To investigate heterogeneity within the tumour additional areas of tumour were sampled from 12 of the choroidal melanoma cases.

For control DNA SKmel-28 and HFF cells were harvested and pelleted by centrifugation at 1500 **g**, 4°C, for 10 min, and washed with PBS. The cells were incubated for 3 h at 50°C in 1 volume lysis solution, (100 mM Tris pH 7.4, 100 mM EDTA pH 8.0, 1% SDS, 1 mg ml^−1^ proteinase K). The lysate was treated by phenol and chloroform extraction, and the aqueous layer centrifuged at 12 000 **g** for 15 min at 4°C. After isopropanol/0.2 M NaCl precipitation at room temperature for 10 min, the sample was centrifuged at 12 000 **g** for 10 min at 4°C. Pellets were washed three times with 75% ethanol, air dried, and dissolved in H_2_O.

### Polymerase chain reaction

#### Primary PCR

Primary PCR amplification reactions were performed in a 25 *μ*l reaction using ReddyMix (ABgene, Epson, UK) containing 5 pmol of forward (5′-TCATAATGCTTGCTCTGATAGGA-3′) and reverse (5′-GGCCAAAAATTTAATCAGTGGA-3′) primer and 5 *μ*l of gDNA template isolated from tumour cells. Reactions consisted of an initial denaturation at 95°C for 3 min followed by 28 cycles of denaturation at 95°C for 20 s, annealing for 30 s at 55°C and extension at 72°C for 45 s. A final extension was carried out at 72°C for 10 min. Three reactions were carried out per DNA extraction.

#### Secondary PCR

Secondary PCRs were performed in a 20 *μ*l reaction using ReddyMix (ABgene) containing 5 pmol of mutation-specific forward (5′-GATTTTGGTCTAGCTACACA-3′) primer, which was optimised to anneal only at the mutation site, if present. Also included were 5 pmol of wild-type forward primer (5′-GCTCTGATAGGAAAATGAGATC-3′) and reverse primer (5′-GTGGAAAAATAGCCTCAATTC-3′), with 2 *μ*l of template from the primary PCR. Reactions consisted of an initial denaturation at 95°C for 3 min followed by 35 cycles of denaturation at 95°C for 20 s, annealing for 30 s at 50°C and extension at 72°C for 45 s. A final extension was carried out at 72°C for 10 min. All reactions were carried out in triplicate.

### Polyacrylamide Gel Electrophoresis

Precast polyacrylamide gels (6%) (Invitrogen, Paisley, UK) were immersed in TBE buffer. Samples were loaded on to the gel and electrophoresed for 1 h at 100V.

### Silver staining

Following electrophoresis the gels were immersed in fixing solution (10% ethanol, 0.007% acetic acid) for 5 min. A further 50 ml of fixing solution containing 0.2 g silver nitrate was added, and gels stained for 10 min. Gels were washed two times in de-ionised water for 20 s and 1 min, respectively. The gel was developed by immersing it immediately in 150 ml of 0.75 M NaOH containing 0.007% formaldehyde until bands appeared. Finally, the gel was rinsed in de-ionised water and washed two times in fixing solution. A band of 200 nucleotides (nt) was obtained in each sample representing the wild-type gene. A further band at 100 nt was present in cases that contained the T1799A mutation. When the wild-type reverse primer was used the second band was approximately 140 nt. In addition, in reactions from DNA extracted from archival paraffin-embedded sections, occasional additional bands were present. This artefact was considered unavoidable as DNA extracted from the paraffin tissue is highly fragmented, and some of the small fragments can lead to self-priming within the reaction.

### Statistical analysis

For choroidal melanoma the relationship of known survival time with *BRAF* mutation, sex, cell type and vascular loops was tested one at a time by a Kaplan–Meier analysis and the relationship of survival time with age and tumour diameter were tested using the ‘Regression with Life Data’ function of Minitab 13.1 (Minitab Inc., State College, PA, USA). The relationship between known survival time and the other variables was also considered in a single multivariate survival analysis. When several tumour areas were sampled from a single tumour association with cell type were assessed by Fisher's exact test.

It was not possible to do this analysis for ciliary body melanoma due to the smaller number of cases and absence of survival data. The relationship between age and the presence of the *BRAF* mutation was assessed by a two-tailed 2-sample *t*-test.

## Results

### SKmel-28 and HFF

**Figure 1:**
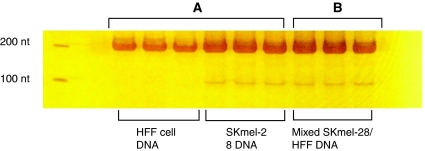
Silver-stained polyacrylamide gel showing a single band at 200 nt for human foreskin fibroblast (HFF) DNA (**A**) and an additional band at 100 nt for SKmel-28 cell DNA (**A**). Mixed DNA from HFF and SKmel-28 (**B**) also shows molecular markers at 200 and 100 bp respectively.

Positive and negative *BRAF* mutation controls were used to ensure the optimisation of the method. Two products of 200 and 100 base pairs (bp) were obtained for SK-mel28, which contains the T1799A *BRAF* mutation and one product of 100 bp for HFF negative control (Figure 1). Identical results were obtained in replicate experiments including titration and kinetic analysis, covering 36 replicates (see Figure 1) Mixing DNA from SKmel-28 and HFF showed that the second (100 bp) band, indicating the presence of the mutation, could still be identified in addition to the wild-type product of 200 bp (Figure 1), when sampling a mixture of one SKmel-28 cell in 100 HFF cells.

### Choroidal and ciliary body melanomas

The T1799A mutation was identified in 4 of the 20 ciliary body melanomas studied and in 11 of the 30 choroidal melanomas examined.

**Figure 2:**
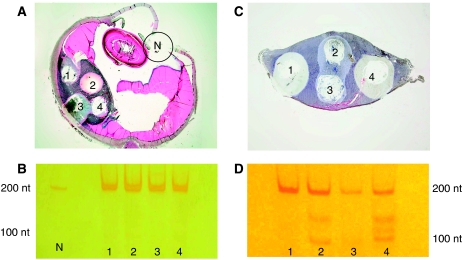
(**A**) Tissue section of enucleation specimen after dissection of four tumour areas (1–4) and one normal tissue area (N). (H&E, magnification × 1.25). (**B**) Silver-stained polyacrylamide gel showing a single band at 200 bp in the four corresponding tumour samples and in the normal tissue sample indicating lack of the T1799A point mutation in this case. (**C**) Tissue section of local resection specimen after dissection of four tumour areas (1–4). (H&E, magnification × 2). (**D**) Silver-stained polyacrylamide gel showing an additional band at 100 nt in samples 2 and 4 indicating the presence of the mutation in these samples. There is an additional band between 200 and 100 nt representing a PCR artefact. Samples 1 and 3 show only one band at 200 nt indicating lack of the mutation.

In addition, sampling of several different areas was undertaken in the 11 positive cases of the choroidal melanoma cases to investigate potential heterogeneity of the T1799A mutation within the tumour sample. Between three and six areas were sampled depending on tumour size (Figure 2; Table 1). Five of the 11 cases contained the *BRAF* mutation in all areas sampled. In four out of the 11 cases the mutation was present in one sampled area only. In two cases the mutation was present in two sampled areas.

### Clinical and pathological details

#### Ciliary body

BRAF-positive cases: Of the four positive cases out of 20 there were three enucleation specimens and one local resection specimen from two men and two women with an average age of 33.5 years (range, 16 to 48 years). Two of the four patients were alive and well, one had died from an unrelated cause. We were unable to obtain clinical follow up information on the fourth patient. The pathology report and original sections were available for review in all cases. The average largest dimension of the tumour was 15.5 mm (range, 10–20 mm). Three tumours were composed of spindle cells and one was of mixed spindle and epithelioid cells. Three of the tumours contained closed vascular loops.

BRAF negative cases: Of the 16 cases out of 20 negative cases there were eight enucleations and eight local resection specimens from seven men and nine women with an average age of 59.6 years (range, 30–80 years). One patient had died of metastatic disease and six patients were alive and well at last follow up. We were unable to obtain clinical follow up information on the remaining nine patients. The pathology report and original sections were available for review in all cases. The average largest dimension of the tumour was 12.7 mm (range, 5–22 mm). Seven tumours were composed of spindle cells and nine were mixed spindle and epithelioid cells. Seven tumours contained closed vascular loops.

The clinical and pathological details are summarised in Table 2. There were no convincingly statistically significant associations between the presence of the *BRAF* mutation and clinical and pathological features.

#### Choroid

BRAF-positive cases: Of the 11 positive cases out of 30 there were six enucleations and five local resection specimens from six women and five men with an average age of 51 years (range, 42–69 years). Clinical follow up was available in all cases. Eight of the patients had died from metastatic tumour and three were alive and tumour free at last follow up. The pathology report and original sections were available for review in all cases. The average largest dimension of the tumour was 14.5 mm (range 10–18 mm). Five tumours were composed of spindle cells, one of epithelioid cells and five were mixed spindle and epithelioid cells. Nine tumours contained closed vascular loops.

BRAF-negative cases: Of the 19 positive cases out of the 30 negative cases there were 12 enucleations and seven local resection specimens from 11 women and eight men with an average age of 57.4 years (range, 19–75 years). Clinical follow up was available in all cases. Eight of the patients had died from metastatic tumour and 11 were alive or had died from other causes. The pathology report and original sections were available for review in all cases. The average largest dimension of the tumour was 14.7 mm (range, 8–23 mm). Eleven of the tumours were composed of spindle cells and eight were of mixed spindle and epithelioid cells. Twelve tumours contained closed vascular loops.

**Figure 3:**
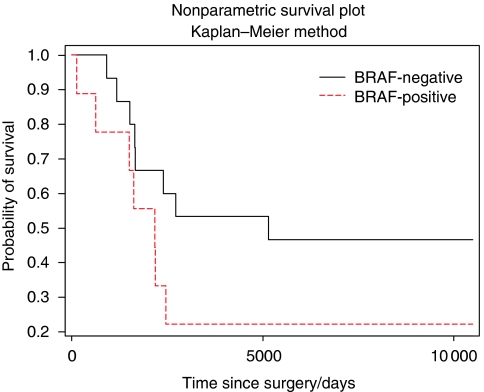
Kaplan–Meier survival curve showing the relationship between time as surgery and survival rate for patients with tumours with and without the *BRAF* mutation.

The clinical and pathological details are summarised in Table 2. The Kaplan–Meier survival curve for patients with tumours with and without the *BRAF* mutation is shown in Figure 3. There was a trend for *BRAF*-positive tumours to show a shorter survival but this was not statistically significant. There were no statistically significant associations between any other clinical or pathological characteristics and the presence of the *BRAF* mutation. Furthermore, in tumours where several areas of the tumour were dissected there was no association between cell type in the individual areas and presence of the mutation.

## Discussion

Activating mutations in the *BRAF* gene have been identified in many human cancers, with the highest frequency of mutations found in cutaneous melanomas (Brose *et al*, 2002; Davies *et al*, 2002; Goydos *et al*, 2005). In melanoma, these *BRAF* mutations are found in two small regions of the kinase domain of the BRAF molecule. The predominant mutation occurs in exon 15 of the *BRAF* gene with a single T-to-A substitution at nucleotide 1799, although a smaller number of mutations have also been found in a region of exon 11(Brose *et al*, 2002; Davies *et al*, 2002; Goydos *et al*, 2005). These mutations have been shown to be present in 66 to 80% of cutaneous melanomas and have also been detected in up to 82% of melanocytic nevi(Pollock *et al*, 2003). The mutation has also been reported in 22–40% of conjunctival melanomas and recently our group has identified this mutation in 48% of 19 iris melanomas (Gear *et al*, 2004; Spendlove *et al*, 2004; Henriquez *et al*, 2007). However, there have been several studies, in uveal melanoma including primary and metastatic choroidal and ciliary body melanomas and the *BRAF* mutation has only been identified in one case (Malaponte *et al*, 2006). Despite this apparent lack of the characteristic *BRAF* mutation, inappropriate downstream MAPK component activation has been reported by Weber *et al* (2003) who failed to identify the *BRAF* mutation in 42 primary uveal melanoma but showed immunohistochemical staining for ERK in 86% of these cases. Similarly, Zuidervaart *et al* (2005) found constitutive activation of the MAPK pathway in 11 uveal melanoma cell lines and 19 primary tumours by performing western blot or immunohistochemistry for the various pathway components. However, an activating BRAF mutation was found in only one cell line. Using the more sensitive nested PCR approach we have identified the T1799A *BRAF* mutation in 4 of 20 (20%) ciliary body melanomas and 11 of 30 (40%) choroidal melanomas.

The majority of other studies have used some form of direct sequencing to identify this mutation (Cruz *et al*, 2003; Edmunds *et al*, 2003; Weber *et al*, 2003). Sequencing would not be able to detect mutant alleles present at low frequency because of somatic mosaicism. Although more sensitive techniques including the ligase detection assay and high amplicon melting PCR have been used to identify the *BRAF* mutation in other tissues including cutaneous melanoma they have not been applied to uveal melanoma (Turner *et al*, 2005; Willmore-Payne *et al*, 2005). Recently, Maat *et al* (2008) detected the BRAF mutation in 6 of 45 uveal melanomas using the more sensitive technique of pyrophosphorolysis-activated polymerisation. In this technique a product is amplified only when the mutation is present even in the presence of tens of thousands of wild-type templates. The nested PCR approach described herein was developed using the SKmel-28 cell line, which is known to harbour the T1799A mutation. Consistent results were obtained using this technique and the mutation could also be detected at low frequency when DNA from SKmel-28 was mixed with wild-type DNA extracted from HFF. The robustness of the primers was established by thoroughly testing the annealing specificity of both wild type-specific and mutation-specific primers designed to anneal to the same site. By dilution of the mutated DNA it was estimated that this technique could detect at least one copy number of the mutation in 100 wild-type genes, and is therefore significantly more sensitive than techniques requiring direct sequencing.

The sensitivity of the PCR technique utilised would make it possible to identify mutant DNA in the presence of wild-type DNA. As separate samples from the same tumour did not always reveal the mutant band, this supports the theory that this mutation is only present in some of the tumour cells. Clonal heterogeneity, particularly morphological heterogeneity is well recognised in uveal melanoma (COMS, 1998) and the majority of tumours are composed of variable proportions of epithelioid and spindle-shaped cells. There have been few studies of cytogenetic heterogeneity in uveal melanoma. Sandinha *et al* (2006) described a heterogenous distribution of cells displaying monosomy three in uveal melanoma and Maat *et al* (2007) described areas of unmethylated and methylated *RASSF1a* within individual uveal melanomas. It is therefore plausible that the *BRAF* mutation could also be distributed heterogeneously. To address this problem we studied several separate tumour areas in 11 choroidal melanomas positive for the T1799A mutation. In six of the tumours positive for the *BRAF* mutation it was observed that the mutation was present in some areas of the tumour but not in others. However, it did not show any association with the cell types in the different areas.

Although the results of this study confirm that the *BRAF* mutation is heterogeneously distributed in uveal melanoma the frequency of this mutation still appears considerably lower than in cutaneous melanoma. This may be due to insufficient tumour sampling. Both cutaneous and uveal tumours share a common embryological origin from the neural crest but there are considerable differences between the cutaneous and uveal environment. For example, ultraviolet light is considered to be an important risk factor in cutaneous melanoma. It has also been suggested that exposure to ultraviolet light may be a key factor in melanomas with the T1799A point mutation(Thomas *et al*, 2006). Previous research has also shown that the *BRAF* mutation frequency is lower in melanoma arising in sites protected from sun exposure compared with those from sun-exposed sites(Cohen *et al*, 2004). Although it is recognised that the *BRAF* mutation is not a UV-signature mutation, it has been suggested that it could still arise due to error-prone reduplication of UV-damaged DNA (Thomas *et al*, 2006). UV exposure is not considered a major factor in choroidal melanoma and this may in part explain the lower frequency of the mutation in the cases studied. An alternative explanation is that this *BRAF* mutation is an infrequent event in uveal melanoma and the observed, inappropriate downstream activation of the MAPK component is due to genetic alterations in other components of this pathway.

In conclusion, we have shown that the *BRAF* mutation is present in a proportion of posterior uveal melanomas and that within these tumours the distribution of this mutation is heterogeneous. This does not appear to carry any prognostic significance but may provide an explanation for the observed upregulation of the MAPK pathway in uveal melanoma.

## Figures and Tables

**Table 1:** The number of mutated and non-mutated tumour areas in cases with the T1796A *BRAF* mutation

	**Number of tumour areas dissected**		
**Mutation-positive cases**	**With the *BRAF* mutation**	**Without the *BRAF* mutation**	**Percentage of areas with mutation (%)**
			
A	1	5	16.67
B	1	4	20.0
C	2	4	33.33
D	1	4	20.0
E	1	2	33.3
F	2	2	50.0
Total	8	21	38.10

**Table 2:** Summary of clinical and pathological details for ciliary and choroidal melanomas with and without the *BRAF* mutation

	**Average age (range)**	**Sex (F : M)**	**Average largest tumour dimension (range, mm)**	**Cell type (S:E:M)**	**Vascular loops (Y:N)**	**Metastases Y:N:not known**	**Follow-up range (months)**
*Ciliary Body*							
BRAF mutation	33 (16–48)	9F:7M	15.5 (10–20)	3:0:1	3:1	0:3:1	N/A
BRAF wild type	59 (30–80)	2F:2M	12.7 (5–22)	7:0:9	7:9	1:6:9	N/A
							
*Choroid*							
BRAF mutation	51 (42–69)	6F:5M	14.5 (10–18)	5:1:5	9:2	8:3:0	4–337
BRAF wild type	57 (19–75)	11F:8M	14.7 (8–23)	11:0:8	12:7	8:11:0	30–344
